# Supplementary material for: A fault-intrusion-tolerant system and deadline-aware algorithm for scheduling scientific workflow in the cloud
Source: PeerJ Comput Sci. 2021 Nov 2;7:e747. doi: 10.7717/peerj-cs.747 (PMC8576554; doi:10.7717/peerj-cs.747)
Supplement: Supplemental Information 6 [file peerj-cs-07-747-s006.zip › Myproject1.1/docs/org/cloudbus/cloudsim/examples/CloudSimExample2.html]

CloudSimExample2


JavaScript is disabled on your browser.


- Overview
- Package
- Class
- Use
- Tree
- Deprecated
- Index
- Help

- Prev Class
- Next Class

- Frames
- No Frames

- All Classes

- Summary:
- Nested |
- Field |
- Constr |
- Method

- Detail:
- Field |
- Constr |
- Method


org.cloudbus.cloudsim.examples

## Class CloudSimExample2

- java.lang.Object
- - org.cloudbus.cloudsim.examples.CloudSimExample2

- ---

    

  ```
  public class CloudSimExample2
  extends java.lang.Object
  ```

  A simple example showing how to create
  a datacenter with one host and run two
  cloudlets on it. The cloudlets run in
  VMs with the same MIPS requirements.
  The cloudlets will take the same time to
  complete the execution.

- - ### Constructor Summary

    Constructors

    Constructor and Description

    `CloudSimExample2()`
  - ### Method Summary

    Methods

    | Modifier and Type | Method and Description |
    | --- | --- |
    | `static void` | `main(java.lang.String[] args)` Creates main() to run this example |

    - ### Methods inherited from class java.lang.Object

      `clone, equals, finalize, getClass, hashCode, notify, notifyAll, toString, wait, wait, wait`

- - ### Constructor Detail


    - #### CloudSimExample2

      ```
      public CloudSimExample2()
      ```
  - ### Method Detail


    - #### main

      ```
      public static void main(java.lang.String[] args)
      ```

      Creates main() to run this example


- Overview
- Package
- Class
- Use
- Tree
- Deprecated
- Index
- Help

- Prev Class
- Next Class

- Frames
- No Frames

- All Classes

- Summary:
- Nested |
- Field |
- Constr |
- Method

- Detail:
- Field |
- Constr |
- Method
